# Vegetation structure and ground cover attributes describe the occurrence of a newly discovered carnivorous marsupial on the Tweed Shield Volcano caldera, the endangered black‐tailed dusky antechinus (*Antechinus arktos*)

**DOI:** 10.1002/ece3.6045

**Published:** 2020-02-12

**Authors:** Caitlin E. Riordan, Coral Pearce, Bill J. F. McDonald, Ian Gynther, Andrew M. Baker

**Affiliations:** ^1^ Earth, Environmental & Biological Sciences Science & Engineering Faculty Queensland University of Technology Brisbane Qld Australia; ^2^ Department of Environment and Science Queensland Herbarium Toowong Qld Australia; ^3^ Threatened Species Unit Department of Environment and Science Bellbowrie Qld Australia; ^4^ Biodiversity and Geosciences Program Queensland Museum South Brisbane Qld Australia

**Keywords:** conservation, Dasyuridae, endangered, habitat characteristics, high altitude, range restricted

## Abstract

The black‐tailed dusky antechinus (*Antechinus arktos*) is a recently discovered, endangered, carnivorous marsupial mammal endemic to the Tweed Shield Volcano caldera, straddling the border between Queensland and New South Wales in eastern Australia. The species' preference for cool, high‐altitude habitats makes it particularly vulnerable to a shifting climate as these habitats recede. Aside from basic breeding and dietary patterns, the species' ecology is largely unknown. Understanding fine‐scale habitat attributes preferred by this endangered mammal is critical to employ successful conservation management. Here, we assess vegetation attributes of known habitats over three sites at Springbrook and Border Ranges National Parks, including detailed structure data and broad floristic assessment.

Floristic compositional assessment of the high‐altitude cloud rainforest indicated broad similarities. However, only 22% of plant species were shared between all sites indicating a high level of local endemism. This suggests a diverse assemblage of vegetation across *A. arktos* habitats.

Habitat characteristics were related to capture records of *A. arktos* to determine potential fine‐scale structural habitat requirements. Percentage of rock cover and leaf litter were the strongest predictors of *A. arktos* captures across survey sites, suggesting a need for foraging substrate and cover. Habitat characteristics described here will inform predictive species distribution models of this federally endangered species and are applicable to other mammal conservation programs.

## Graphical abstract

The black‐tailed dusky antechinus (*Antechinus arktos*) is a recently discovered, endangered carnivorous marsupial mammal endemic to the border between Queensland and New South Wales in eastern Australia. The species' preference for cool, high‐altitude habitats makes it particularly vulnerable to a shifting climate. Here, we assess vegetation attributes of known habitats over three sites at Springbrook and Border Ranges National Parks, including detailed structure data and broad floristic assessment.

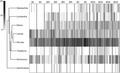

## 1 INTRODUCTION

Research indicates climate change is increasing atmospheric temperatures, in turn reducing the home ranges of many animal species, particularly those confined to montane habitats (Colloff et al., 2016; Gray, Baker, & Firn, 2017; IPCC, 2014). With increasing change, conservation management priorities for threatened and rare species within these habitats must be flexible and strategically tailored to meet the needs of the species in question, maximizing the chances of successful preservation (Ceballos et al., 2015). Both habitat and vegetation structure play a vital role for many species in both evolutionary and ecological functioning and processing (Gibson, Blomberg, & Sedinger, 2016). For endangered or rare species, this notion necessitates a specific understanding of how habitat structure and vegetation composition may impact animal occurrence and the habitat selection by species (Dinsmore, White, & Knopf, 2002; Gibson et al., 2016).

Recent studies describing critical habitat of threatened or newly discovered species often make use of aerial imagery and geographic information systems (GIS) to identify areas of known occurrence, areas of conservation concern, or to establish potential suitable habitat (Reza, Abdullah, Nor, & Ismail, 2013; Turner, Douglas, Hallum, Krausman, & Ramey, 2004; Zlinszky, Heilmeier, Balzter, Czúcz, & Pfeifer, 2015). Furthermore, some studies use this method exclusively to describe a species' habitat and utilize largely abiotic variables to quantitatively assess habitat preferences (Turner et al., 2004). However, many small mammal species, especially those with limited or patchy distributions, will not necessarily be well‐represented by broad‐scale vegetation assemblages, such as those produced by GIS and aerial photography (Cusack, Wearn, Bernard, & Ewers, 2015; Mason, Firn, Hines, & Baker, 2017; Rowe et al., 2015; Stirnemann, Mortelliti, Gibbons, & Lindenmayer, 2015). Rather, fine‐scale habitat and vegetation attributes that are structurally complex at the microhabitat level may be key drivers of small mammal occurrence within many ecosystems (Stirnemann et al., 2015).

One method of understanding how microhabitats influence abundance and finer‐scale occurrence of mammals adopts a comparison of autecological capture and occurrence data, linked with fine‐scale structural vegetation attributes. Various studies have considered such relationships between microhabitat use and structure for small mammals (e.g., Cusack et al., 2015; Diffendorfer, Gaines, & Holt, 1999; Kelly & Bennett, 2008; Morris, 1984; Santos, Thorne, & Moritz, 2015; Smith, Means, & Churchill, 2017; Stokes, Pech, Banks, & Arthur, 2004). These studies provide important information about microhabitat preference and use, which is invaluable for the development of conservation programs.

Such information was assessed for a recently discovered, endangered species of Australian carnivorous marsupial: the silver‐headed antechinus (*Antechinus argentus*). The species' known habitats were found to be complex in structure, with individuals utilizing large logs and Johnson's grass‐tree (*Xanthorrhoea johnsonii*) as refugia, along with leaf litter, presumably for foraging purposes (Mason et al., 2017). For *A. argentus*, this research filled a fundamental gap in ecological knowledge with benefits for conservation programs being developed for this species.

Several other new *Antechinus* species have recently been described and their ecology is poorly understood (see Baker, Mutton, Mason, & Gray, 2015).

In May 2018, one other species was listed as endangered under federal legislation (EPBC act 1999), namely the Black‐tailed dusky antechinus (*Antechinus arktos*) from the Tweed Shield Volcano caldera, straddling the Queensland/New South Wales border on mid‐eastern Australia (Baker, Mutton, Hines, & Dyck, 2014). This species is the focus of the present study.

Forested environments, such as the Gondwanan rainforest relics of the Tweed Shield Volcano caldera, harbor much of the endemic and endangered faunal species occurring in Australian states (Figgis, 2000; Scarlett et al., 2015). Since description of *A. arktos* in 2014, the species has been listed as endangered under both state and federal legislation, due to its highly fragmented, high‐altitude habitat, limited potential for distribution on the caldera, extremely low apparent abundance, and a range of threats exacerbating these problems (Baker et al., 2014; Gray, Baker, et al., 2017; Gray, Burwell, & Baker, 2016; Gray, Dennis, & Baker, 2017). However, these factors remain poorly understood and need urgent research to ensure effective conservation management (Baker et al., 2014; Gray, Baker, et al., 2017; Gray et al., 2016; Gray, Dennis, et al., 2017). Foundational studies have been conducted on the species' taxonomy by Baker et al. (2014) and reproductive biology, population dynamics, and dietary preference by Gray et al. (2016), Gray, Baker, et al. (2017), and Gray, Dennis, et al. (2017). Information on the species' habitat use and preference represents a crucial knowledge gap.

Therefore, the present study aims to produce a fine‐scale habitat description of known *A. arktos* sites and relate these findings to mark–recapture information of the species (see Gray, Baker, et al., 2017). *Antechinus arktos* has apparently retracted altitudinally in recent decades and is threatened with imminent extinction under various climate change scenarios (Baker et al., 2014). A robust knowledge of structural habitat attributes and microhabitat use is paramount to understand how best to conserve *A. arktos* into the future.

Specifically, we will therefore address the following research questions:

1. How do known *A. arktos* sites differ in vegetation structure and composition?
2. How do fine‐scale structural and cover habitat attributes differ between known sites?
3. How do *A. arktos* captures relate to fine‐scale habitat attributes?

## 2 MATERIALS AND METHODS

### 2.1 Study sites

The study sites were established within the World Heritage‐listed Tweed Shield Volcano caldera, a relic from late Tertiary times (23 mya). Sites were based on a previous study of historical capture records by Baker et al. (2014) and parallel research that assessed the present‐day distribution of *A. arktos* (Gray, Baker, et al., 2017; Gray Burwell & Baker, 2016).

The sites lay within three national parks in the region straddling the border between Queensland and New South Wales: Springbrook National Park and Lamington National Park (both in Queensland), and Border Ranges National Park (in New South Wales). Springbrook National Park formed the major component of the study sites, as this area has exhibited the greatest abundance of *A. arktos* in the years since its discovery.

We used two sites within Springbrook National Park: Best of All Lookout (−28.2415°S, 153.2640°E) and Bilborough Court (−28.2341°S, 153.2897°E), both ~950 m above sea level (ASL). The holotype locality for *A. arktos*, Best of All Lookout, is largely comprised of complex notophyll vine forest, Regional Ecosystem (RE) 12.8.5 (Queensland Herbarium, 2016) and simple microphyll fern forest (RE 12.8.6). The site is characterized by the cloud‐stripping stream lily (*Helmholtzia glabberima*) in its gullies, with small stands of a northern outlier of Antarctic beech (*Nothofagus moorei*) at the highest elevations. Best of All Lookout habitat has been largely undisturbed by clearing in recent decades and is a popular tourist attraction. The bitumen access path leading to the lookout marks the Queensland/New South Wales border. The Bilborough Court site is comprised of regenerating complex notophyll vine forest as well as simple microphyll fern forest and is markedly more open than Best of All Lookout. It was most recently partially cleared between 1961 and 1989 (Queensland Government, Q Imagery, 2017). The two Springbrook sites were assessed for both the plant structural comparative analysis and the species composition components of the study.

A third site used in this study was Bar Mountain in Border Ranges National Park (−28.3558°S, 152.9850°E), ~27 km (geodesic distance) from Best of All Lookout at Springbrook National Park. High altitude (900–1,250 m) and broad habitat features suggest suitability for *A. arktos*, but prior to the present study, *A. arktos* had not been found at this site. However, in 2017 a parallel study involving a detection dog, with confirmation by fixed white flash cameras, discovered *A. arktos* at Bar Mountain after previous concerted and targeted efforts over the last decade using traditional live trapping (using Elliott type A traps) had failed (Gray, Baker, et al., 2017; Thomas, Baker, Beattie, & Baker, 2020). Vegetation structure and ground cover data at Bar Mountain were included in the present study as a means of describing habitat structure and incorporating a third comparative location for *A. arktos*.

A fourth site at Toolona Lookout, Lamington National Park (−28.2513°S, 153.1760°E), located ~8 km (geodesic distance) west of the holotype locality, was also selected as the species is known to occur in this location. The site comprised RE 12.8.6 and is ~1,100–1,250 m ASL. Logistical constraints precluded the site being used for comparative vegetation and capture data analysis; thus, the site was assessed only for the species composition component of the present work.

All four known *A. arktos* sites were used for the plant species composition component of the study. Three sites (Best of All Lookout, Bilborough Court, and Bar Mountain) were used to compare habitat structure. Two sites (Best of All Lookout and Bilborough Court) were used for the comparative vegetation/animal capture component of the study. In the latter study component, we assumed that trap position of *A. arktos* related to habitat use, since the animal would have been foraging when it was captured. This enabled us to examine the relationship between density of *A. arktos* with surrounding microhabitat structure and type.

### 2.2 Vegetation surveys

Floristic assessment of the survey sites was conducted by the Queensland Herbarium following the Queensland Government “Basic Site Information” protocol (Eyre et al., 2014). This included a brief habitat description, Regional Ecosystems (RE) classification, broad vegetation grouping, and dominant/characteristic plant species identified.

Surveys assessing habitat structure were conducted at the two Springbrook NP sites and Border Ranges NP site between 14 and 21 July 2017, to determine fine‐scale habitat attributes associated with presence of *A. arktos*. Vegetation was grouped based on functional groups, allowing for comparisons in habitat structure between sites where floristics are expected to vary. Vegetation surveys at Springbrook NP were performed along four pre‐established transects, which had previously been used as trap lines for detection of *A. arktos* (see Gray, Baker, et al., 2017). This survey method was adapted from Nguyen, Kuhnert, and Kuhnert (2012) and Bonham (2013) and utilized 5‐meter radius circular quadrants at every third trap of the grid lines. In each case, the specific trap location was the centerpoint of the circular quadrant. A total of 32 (8 plots along 4 transects) trap locations were surveyed at each of the three sites, equating to a total of 96 (32 × 3 sites) points for the entire study. At each site, total cover was estimated for each of the tree and shrub layers, plus the lianas (vines), palms, and tree ferns, while estimates of wait‐a‐while (*Calamus muelleri*), rainforest lomandra (*Lomandra spicata*), stream lily (*Helmholtzia glaberrima*), and rock cover were recorded as a percentage of the entire circular quadrat. Ground cover was assessed using a 1 m^2^ quadrat randomly placed three times within each circular quadrat survey areas. Percentage of each ground covered by each functional group (forbs, bare soil, rock, moss/lichen, grass, ferns, leaf litter and roots and logs) was estimated. As functional groups could overlap, the total quadrat estimate could exceed 100% (Daubenmire, 1959).

### 2.3 Animal trapping

Live capture (Elliott) trapping was conducted prior to the present study during 2014 and 2015 at the two known *A. arktos* sites at that time: Best of All Lookout and Bilborough Court, in Springbrook NP, Queensland. Gray, Baker, et al. (2017) utilized a trapping grid of 200 traps per site, consisting of four equally spaced (20 ms apart) parallel transects of 25 trap stations (each double‐trapped, equaling 50 traps per transect). In each transect, trap stations were uniformly distributed, 8 meters apart. Equal trapping effort was conducted at each of the two sites monthly between April and October of both years. In each month, trapping was conducted over six nights across the two sites, with traps open on alternate nights at each site, equating to three nights of trapping per site each month. This totaled 16,800 trap‐nights (600 trap‐nights × 2 sites × 7 months × 2 years). Gray, Baker, et al. (2017) acknowledged the delineated grid at each site covered a small area. However, both sites are mountainous with difficult access, and the grids covered a large proportion of both accessible and suspected suitable, highest altitude habitat for the species. For more specific information about mammal surveying techniques, see Gray, Baker, et al. (2017).

In the present study, we utilized the existing trapping grid of Gray, Baker, et al. (2017) to facilitate direct comparison between animal capture and vegetation surveys. We conducted vegetation surveys at every third trap location along each trap line. The total number of captures for that trap and the two surrounding traps from the same line were included to determine a range in which individuals may occur.

Although the species is now known from four sites, detailed trapping data used in the present study were only available from the two sites included by Gray, Baker, et al. (2017).

### 2.4 Statistical analyses

Difference between floristic assemblages of the four known *A. arktos* sites was determined using a similarity dendrogram. This allowed for relationships between sites based on vegetation species to be assessed as a percentage of relatedness, grouping sites of closer species assemblage together (Anderson, Gorley, & Clarke, 2008; Field, Clarke, & Warwick, 1982).

A nonmetric multidimensional scaling (nMDS) plot was constructed providing an ordination (with sites represented as points in multidimensional space) of differences between vegetation species assemblages among the four sites (Anderson et al., 2008). Both analyses used Bray–Curtis similarity matrices (Bray & Curtis, 1957).

To analyze differences in vegetation structure, a fourth‐root transformation was required to reduce the influence of dominant functional groups (Gray et al., 2016; Smith et al., 2017). A resemblance matrix was produced using a Bray–Curtis similarity statistic (Anderson et al., 2008; Bray & Curtis, 1957). To assess difference in vegetation structure, a multivariate approach was used. A PERMANOVA (permutated MANOVA) was conducted. A subsequent pairwise post hoc test was conducted to determine finer‐scale relationships.

To relate vegetation structural differences between sites at transect and plot level to *A. arktos* captures, nMDS plots were created. The vegetation survey plots were treated as nested within transects and transects nested within sites. Each point of the nMDS demonstrates the vegetation structure with the additional overlay called a “bubble” representing *A. arktos* captures. The size of the bubble represents the number of *A*. *arktos* captures (recaptures included) within the vegetation survey plot.

As the vegetation surveys were conducted at every third trap point, we considered *A. arktos* captures from the surveyed plot, plus one trap point either side of the center trap, assuming *A. arktos* individuals utilize proximate ranges in their habitat (as per Mason et al., 2017; Pearce, 2016). Shade plots and dendrograms were created (Anderson et al., 2008) to visualize structural similarities or differences between the three sites for vegetation structure. A square‐root transformation was performed, factored by transect.

Ground cover was assessed based on three replicates of quadrant estimates of cover per 5 m radius plot. The results of the three quadrates surveyed were averaged for each cover type in the analysis. The data were then fourth‐root transformed, to reduce potential over‐contribution of dominant attributes of ground cover. A resemblance matrix was produced using the Bray–Curtis similarity statistic (Bray & Curtis, 1957). To test the difference in ground cover attributes between the three sites and between the Springbrook NP sites alone, multivariate analyses were performed (Anderson et al., 2008). A principal component analysis (PCA), rather than nMDS, was conducted as the data were recorded as percentages (rather than counts; Anderson, 2001; Anderson et al., 2008; Clarke & Gorley, 2015; Mason et al., 2017; McArdle & Anderson, 2001). As with previous analysis, survey plots were treated as nested within transects and transects nested within sites. This analysis aimed to visualize the difference in ground cover for Springbrook NP sites in relation to *A. arktos* capture rates and so a “bubble” overlay was used (as described previously). A shade plot and dendrograms were created to visualize structural similarities or differences between the three sites for ground cover. This followed a square‐root data transformation prior to analysis. All aforementioned analyses were conducted using PRIMER 7 (Anderson et al., 2008).

To determine relative importance of habitat attributes at Springbrook NP to *A. arktos* captures, boosted regression tree (BRT) models were used. This allowed the measurement of both vegetation structure and ground cover to determine the relative importance each predictor variable has on the response variable (Elith, Leathwick, & Hastie, 2008). The BRT approach is an ensemble method that involves fitting one most parsimonious model using binary splits that are dependent on predictor variables. This divides the observed data into groups with greater similarity to the data of the response variable, namely *A. arktos* capture rates. Boosting then uses repetitions to fit models to the data in stages, permitting a range of predictor variables to be used within one analysis (Elith et al., 2008). A Poisson distribution was utilized as the capture rate from each trap was counted in whole numbers rather than binary (Elith et al., 2008). Models were performed on the vegetation structure data and the ground cover (%) attributes. These models were performed using R statistical computing program version 3.2.2 adopting the “gbm” package version 2.1.1 (RStudio Team, 2015).

## 3 RESULTS

### 3.1 Plant species assemblages

A total of 95 plant species were recorded, with 22% being present at all four sites. Bar Mountain (62 species) and Best of All Lookout (61) exhibited the greatest species richness, while Bilborough Court (47) and Toolona Lookout (41) were lower. A total of 18 shrub species were recorded across the four sites, with five representing dominant/characteristic species across three or more sites (see Appendix A). Thirty‐two tree species were recorded, with 11 species found in at least three sites. Vines, lianas, and epiphytes (including climbing and epiphytic ferns) were grouped together with a total of 26 species, 15 of which were characteristic across three or four sites. Palms comprised only three of the plant species from the four sites; these included wait‐a‐while (*Calamus muelleri*), which was only found in the two Springbrook National Park sites. Nonepiphytic ferns, tree ferns, forbs, grasses, and stream lily (*Helmholtzia glabberima*) made up the final component of species richness, represented by a total of 15 species, with seven of those being characteristic across three or four sites.

Of the four sites surveyed for plant species assemblages, Best of All Lookout and Bilborough Court were most similar in their assemblages (~75%), followed by Bar Mountain (~70%, clustered with Best of All/Bilborough) and then Toolona Lookout (~50%, clustered with Bar Mountain and Best of All/Bilborough). Geographic separation of the sites reflects this trend.

### 3.2 Habitat structure

**Figure 1:**
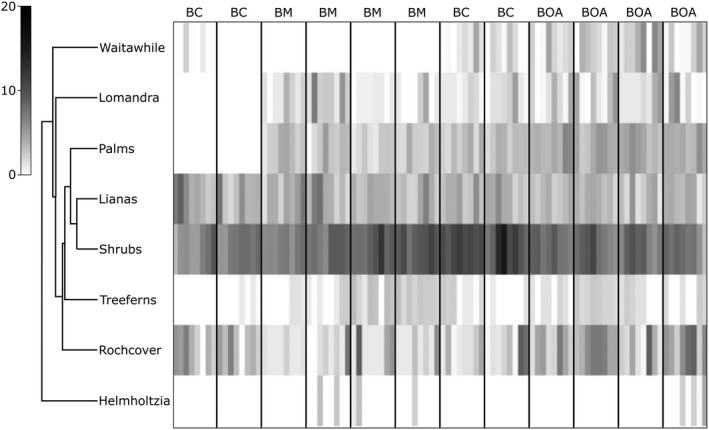
Shade plot dendrogram indicating the relative contribution by abundance of structural vegetation variables at Best of All Lookout (BOA), Bilborough Court (BC), and Bar Mountain (BM). Variables with higher abundance are shown in dark shades, while lower abundance is shown in lighter shades. Legend (left) indicates coloration of percentages

**Figure 2:**
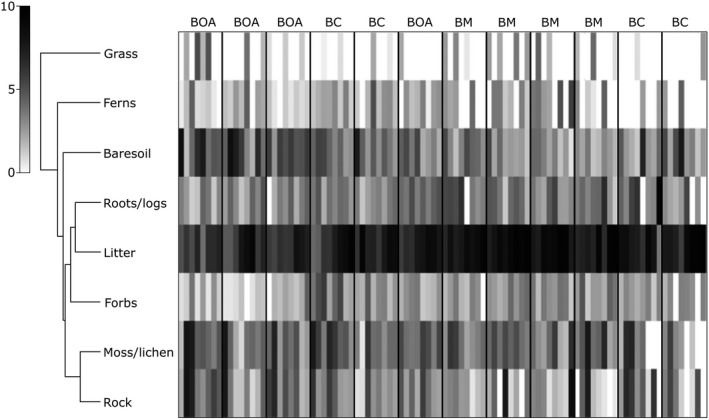
Shade plot dendrogram indicating the relative contribution by abundance of percentage ground cover variables at Best of All Lookout (BOA), Bilborough Court (BC), and Bar Mountain (BM). Variables with higher abundance are shown in dark shades, while lower abundance is shown in lighter shades. Legend (left) indicates coloration of percentages

A multivariate analysis of vegetation structure and percentage ground cover between the three survey sites showed significant differences between all pairwise comparisons. All three combinations were significantly different for vegetation structure (PERMANOVA, *t* = 4.42, *p* = .0001 for Best of All × Bilborough; PERMANOVA, *t* = 4.65, *p* = .0001 for Best of All × Bar Mountain; PERMANOVA, *t* = 3.59, *p* = .0001 for Bilborough × Bar Mountain). A shade plot denoting similarity in vegetation structure and ground cover between the sites surveyed were created (see Figures 1 and 2).

Ground cover attributes were also found to be significantly different between Best of All Lookout and the other two sites (PERMANOVA, *t* = 2.40, *p* = .0005 for Bilborough Court; PERMANOVA, *t* = 2.93, *p* = .0001 for Bar Mountain), while the least significant difference was between Bilborough Court and Bar Mountain for percentage ground cover (PERMANOVA, *t* = 1.59, *p* = .0406) suggesting these sites differed less in ground cover attributes and structure when compared to other pairwise site combinations.

### 3.3 Vegetation structure in relation to *A. arktos* captures

Multivariate analysis of plant functional groups and *A. arktos* captures across the Springbrook NP sites displayed significant differences between 17 of the 28 pairwise comparisons between transects. Most of the pairwise contributions with significant *p*‐values for vegetation cover (12 out of 17) were different between transects at Best of All Lookout and two transects at Bilborough Court exhibiting no captures of *A. arktos* (see Appendix C for values of each significant pairwise comparison). Results from a multivariate analysis of vegetation structure and ground cover between these sites are also shown in Appendix C.

**Figure 3:**
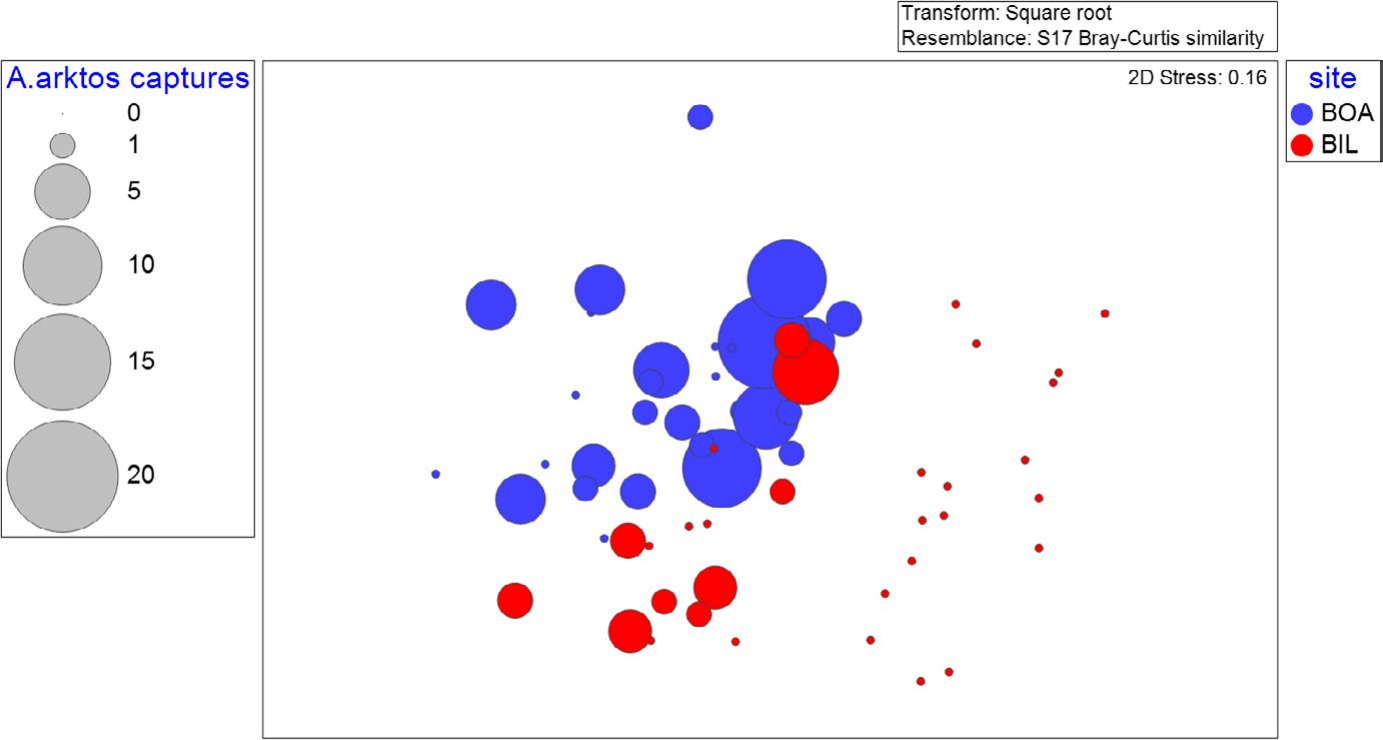
Nonmetric multidimensional scaling (nMDS) bubble plot of vegetation structure through functional groups at each 5 m radius survey transect (BOA = Best of All Lookout, BIL = Bilborough Court). The size of the bubbles denotes the number of *Antechinus arktos* captures during 2014 and 2015 by Gray, Baker, et al. (2017) and placement of bubbles indicates similarity of vegetation structure relative to all other data points. The colors of each bubble indicate the site the animals were captured

The two survey sites at Springbrook National Park were clearly separated based on the nMDS of vegetation and habitat structure, when related to *A. arktos* capture density (Figure 3). Transects where *A. arktos* captures were recorded (shown in “bubbles”) indicated clear similarities in aspects of habitat and vegetation structure, as denoted by the clustering of capture events to the right of the plot. Those that do not have a corresponding capture bubble also tend to be grouped together, clustered to the left of the plot. This suggests less favorable habitat for the target species.

“Bubbles” within Figure 3 from Bilborough Court tend to show fewer captures than many sites within Best of All Lookout, and this reflects overall capture differences between the two sites (82 at Best of All Lookout vs. 21 at Bilborough Court). There is spatial structure in the number of captures, as indicated by the relative size and positioning of bubbles.

**Figure 4:**
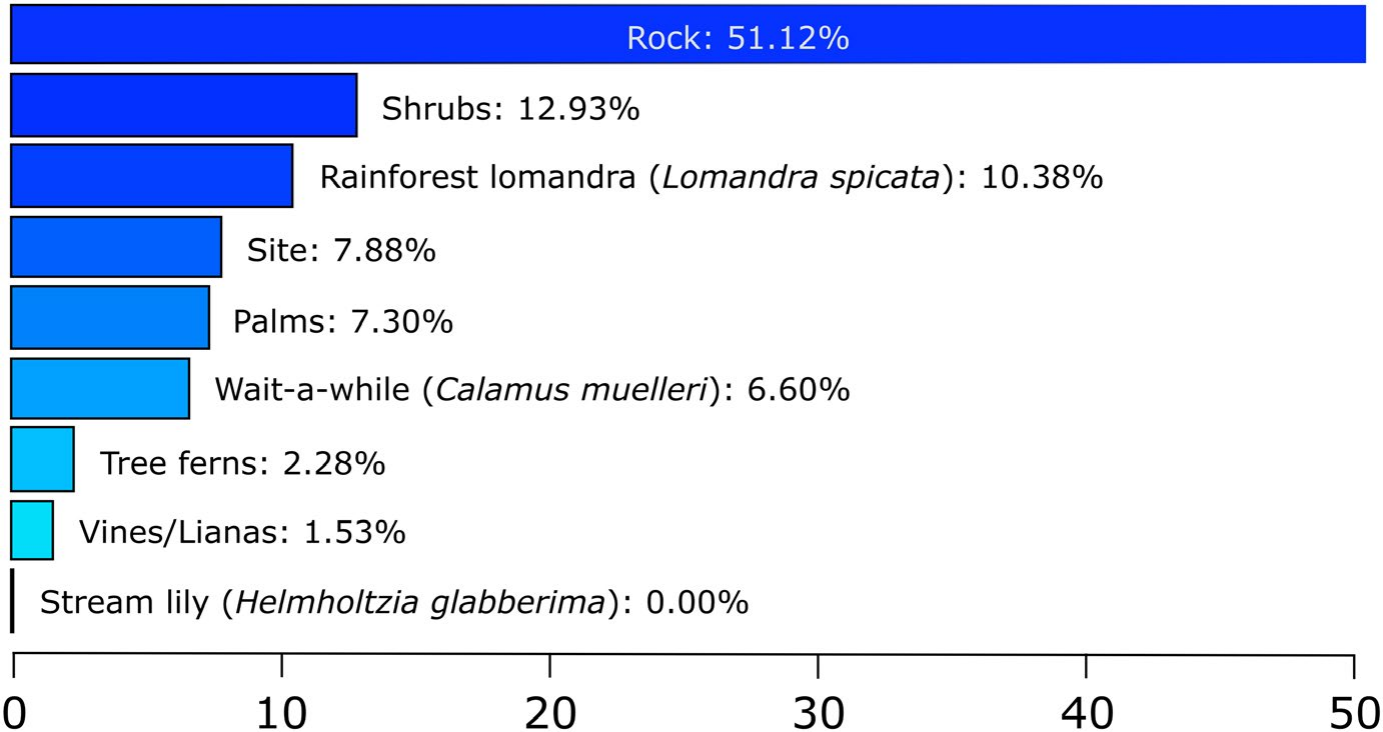
BRT model of vegetation structure through functional groups from Best of All Lookout and Bilborough Court. Relative influence percentages of each variable relating to *A. arktos* capture are indicated on each bar. Developed with cross‐validation on data using 1,200 trees, tree complexity of 2 and learning rate of 0.004

Of the nine vegetation and habitat structure variables assessed, the greatest relative influence when compared to *A. arktos* captures was percentage of rock cover (51.12%), as shown in Figure 4. The presence of shrubs was found to be the next most influential variable (12.93%) as indicated by the model, followed by rainforest lomandra (10.38%). Based on this model, the estimate of correlation between observed and predicted response variables was 57%, suggesting a moderate correlation (Elith et al., 2008).

### 3.4 Ground cover in relation to *A. arktos* captures

Results of multivariate analysis of ground cover characteristics show less statistical variation than those displayed for plant functional groups with ten significant groupings out of the 28 pairwise comparisons between transects. Seven out of 10 significant pairwise comparisons were between transects that had exhibited trap success, compared to those that had no trap success for *A. arktos* (see Appendix C for breakdown of individual significance statistics).

**Figure 5:**
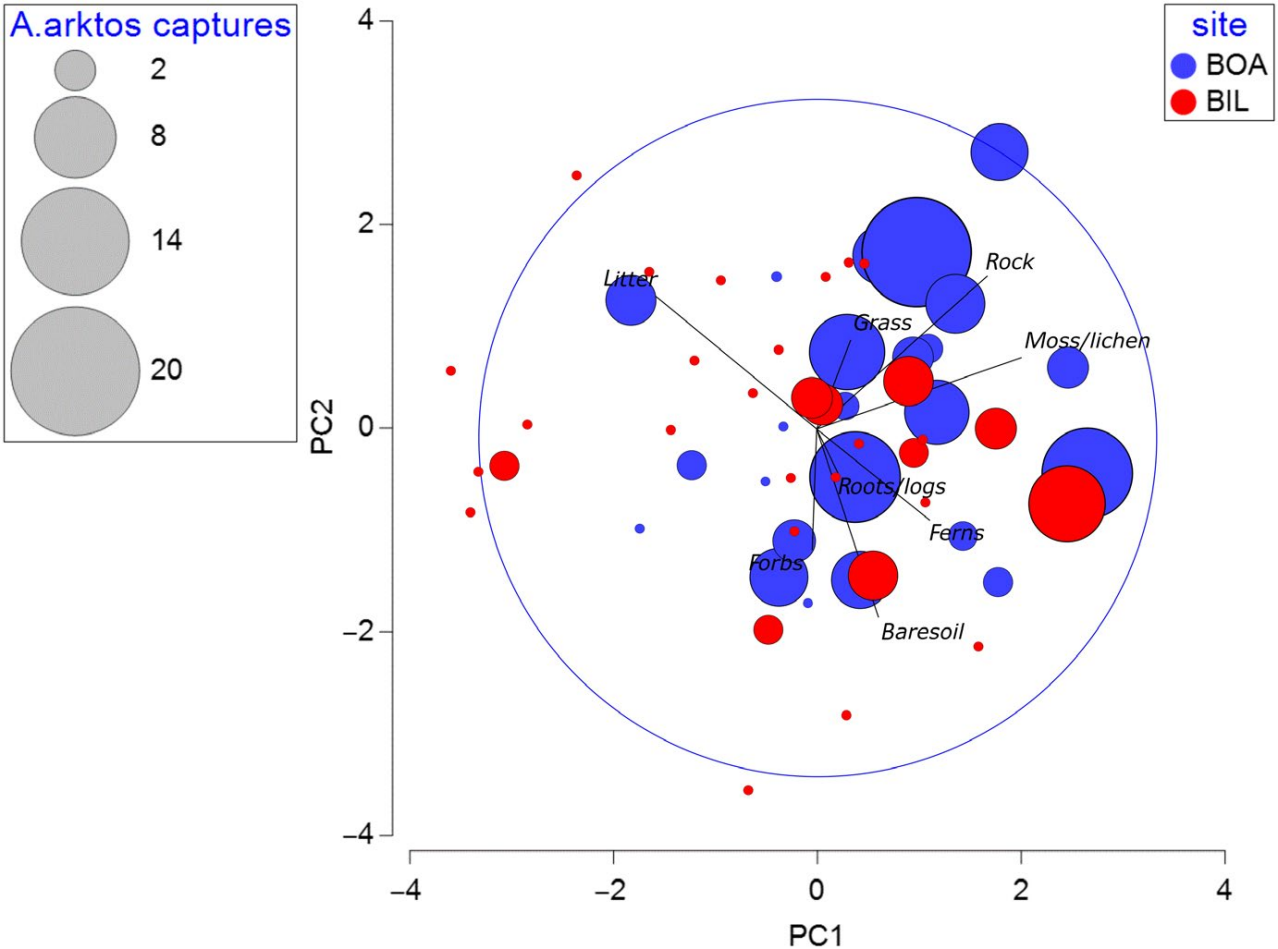
Principal component analysis (PCA) bubble plot of percentage ground cover at Best of All Lookout and Bilborough Court comprised of forbs, bare soil, rocks, moss/lichen, ferns, grass, roots and large logs and leaf litter averaged from three randomly placed 1 m^2^ at each 5 m radius plot. Axes have no physical meaning; they are “principal components” that visualize variation. The size of bubbles denotes the total *A. arktos* captures during 2014 and 2015 by Gray, Baker, et al. (2017)

Percentage of ground cover among the two sites showed some spatial separation when analyzed with principal component analysis (PCA; Figure 5). Bubbles, indicating *A. arktos* capture abundance, were spatially positioned to the right of the plot. This suggests that Best of All Lookout provides the most preferable ground cover habitat for *A. arktos*.

**Figure 6:**
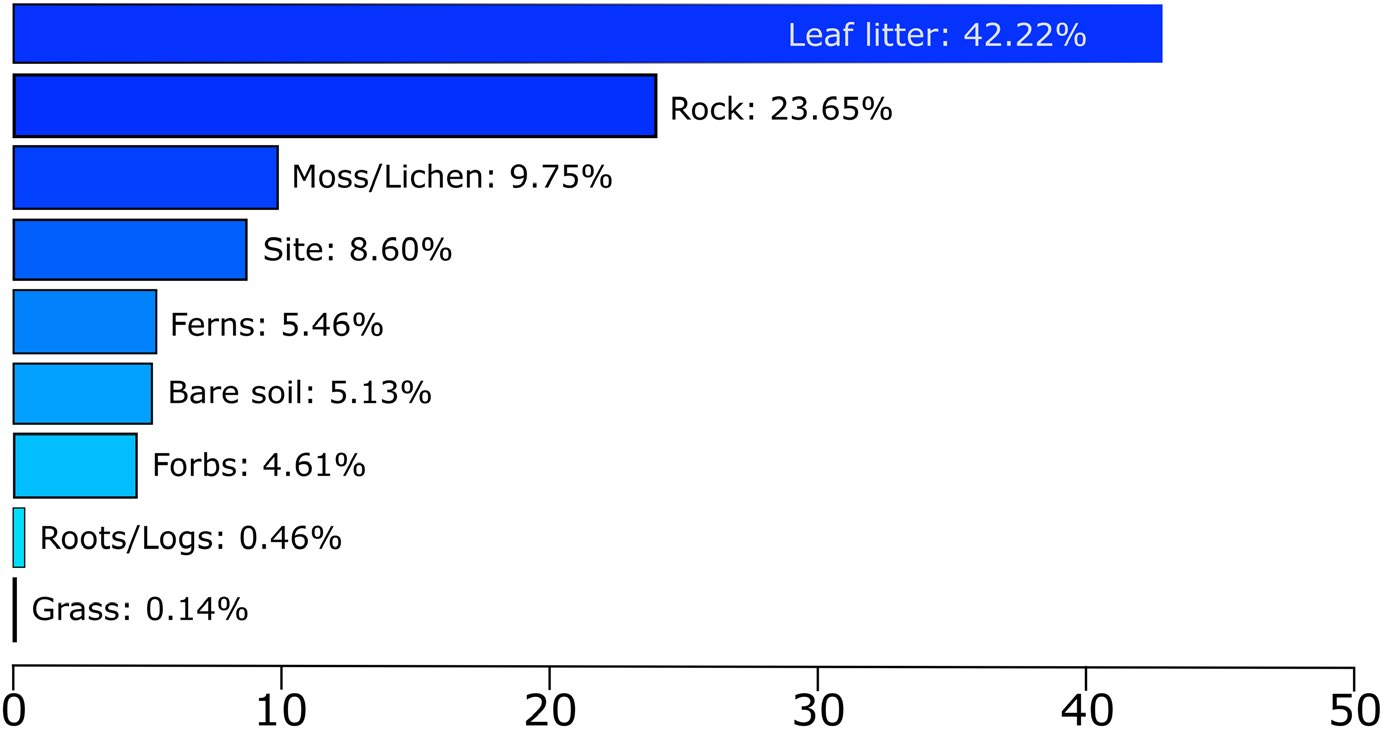
Boosted regression tree (BRT) model of percentage ground cover from Best of All Lookout and Bilborough Court. Relative influence percentages on *A. arktos* capture for each variable are indicated on each bar. The plot was developed with cross‐validation on data using 1,200 trees, with a tree complexity of 2 and learning rate of 0.004

Of the nine variables from the percentage ground cover data set, two variables had high relative influence on capture rates of *A. arktos* at Best of All Lookout and Bilborough Court. The BRT models showed that leaf litter percentage (42.22%) had the greatest relative influence on *A. arktos* incidence, with rock cover percentage (23.65%) having the second greatest relative influence (Figure 6). The correlation between observed and predicted response variables from this BRT was 59%, suggesting a moderate correlation for this model (Elith et al., 2008).

## 4 DISCUSSION

Our research aimed to determine microhabitat use by *A. arktos* across the species' known range. We found that while there were a suite of plant species characteristic of high‐altitude cool temperate rainforest found between the four sites, composition largely varied between sites. We found that fine‐scale habitat attributes differed markedly between the sites surveyed (Figures 1, 2, 4 and 5). Of the habitat structure variables assessed, rock cover and leaf litter were most closely associated with *A. arktos* captures. Each of these major findings is investigated in more detail below.

### 4.1 How do known *A. arktos* sites differ in vegetation structure and composition?

Many of the 22% of species shared among the survey sites, such as golden sassafras (*Doryphora sassafras*), mountain butterfly vine (*Pararistolochia laheyana*), and prickly ash (*Orites excelsus*), are typically associated with high‐altitude cool temperate plant communities and rainforests in eastern Australia (Buchwalder, Samain, Sankowsky, Neinhuis, & Wanke, 2014; Laidlaw, McDonald, Hunter, & Kitching,^2011^; Lowman, 1992). Similarities of floristic assemblages across high‐altitude isolated areas of the Tweed caldera may be the result of volcanic formation of the area and proximate connection within the Tweed volcano prior to its degradation 20–24 million years ago (Graham, 2010; Kooyman, Rossetto, Cornwell, & Westoby, 2011; Weber, VanDerWal, Schmidt, McDonald, & Shoo, 2014).

The majority of vegetation species were site specific. The greatest compositional similarity was found between Best of All Lookout and Bilborough Court, which are also geographically the closest sites. Springbrook National Park was similar to Bar Mountain, which is geographically the furthest from the *A. arktos* holotype locality, Best of All Lookout. Toolona Lookout exhibited the least shared plant species among sites, being most different to the Springbrook and Border Ranges sites.

Erosion of the Tweed Caldera over time has caused geographic isolation, creating microclimates in the highest reaches of the caldera. Climate change has further exacerbated this isolation, resulting in high levels of endemism of both plant and animal species (Weber et al., 2014). A study of endemism patterns and species richness within subtropical Queensland and New South Wales found that the Border Ranges region exhibited largest variation in plant species, containing 78% endemic rainforest plant taxa. It was found that 65% of endemic rainforest plants are found near or within Border Ranges, Springbrook, and Lamington National Parks (Weber et al., 2014). Increased rainfall and co‐occurrence of tropical, subtropical, and temperate plant taxa at Border Ranges NP may explain the increased species diversity and endemism at this site. Limitations in plant species dispersal within rainforests south of the Tweed caldera are the strongest driver of plant species endemism and differing assemblages, even in small study areas (Rossetto, Kooyman, Sherwin, & Jones, 2008). Given the observed high levels of site‐specific endemism particularly at the Border Ranges, Bar Mountain may act as a “climatic refugia” for endemic plant species, protecting those confined to cool temperate rainforest from abrupt climate change (Weber et al., 2014).

### 4.2 How do structural and cover attributes differ between known *A. arktos* sites?

Notably, Bilborough Court and Bar Mountain exhibited the greatest shrub cover with least rock cover, while Best of All Lookout had intermediate shrub cover, the most rainforest lomandra (*Lomandra spicata*) cover, and highest rock cover. For ground cover percentages among the three sites, Bar Mountain and Bilborough Court were similar in exhibiting high leaf litter and low bare soil values, while Best of All Lookout displayed intermediate leaf litter and the highest amount of bare soil (Figures 2 and 3).

Variation in vegetation structure among sites may be related to level of historical disturbance. Disturbances at the sites examined range from low at Best of All Lookout, which is purported to support the highest abundance of *A. arktos* (Gray, Baker, et al., 2017) and is largely preserved, to high at Bilborough Court and Bar Mountain. Both latter sites were logged in the 20th Century (Queensland Government, 2017). More recently, Bar Mountain suffered damage from ex‐tropical cyclone Oswald in 2013 (Smith, 2015). In contrast, Toolona Lookout at Lamington National Park, like Best of All Lookout, supports a higher density population of *A. arktos* (Gray, Baker, et al., 2017) and has suffered only minimal disturbance (Laidlaw, Olsen, Kitching, & Greenway, 2000).

### 4.3 How do *A. arktos* captures relate to fine‐scale habitat attributes?

In Australia's montane cool temperate rainforests, vegetation structure influences the composition, distribution, and abundance of many small mammals (Dickman, 1982; Holland & Bennett, 2007; Mason et al., 2017; Mortelliti, Amori, & Boitani, 2010; Poskitt, Duffey, Barnett, Kimpton, & Muller, 1984; Stirnemann et al., 2015; Stokes et al., 2004). Heterogeneity at the finest‐scale within such habitats may also be a determining factor of mammalian occurrence (Holland & Bennett, 2007; Stirnemann et al., 2015). We found significant difference in vegetation structure between the two sites in Springbrook NP.

Of the two sites, *A. arktos* was most frequently captured at Best of All Lookout. Vegetation structure differed between these two sites, driven by decreased vegetation complexity such as less lomandra, palms, and vines as well as tree ferns at Bilborough Court. Notably, transects at Bilborough Court that showed this vegetation characteristic had no recorded *A. arktos* captures, while the inverse was true at transects with the highest levels of *A. arktos* captures at Best of All Lookout. The presence of ferns and grasses (predominantly rainforest Lomandra) was positively related to *A. arktos* captures.

Variation in captures within both sites was also observed, with a trend of most captures being recorded at sites of high vegetation structural and ground cover diversity.

Taken together, these results suggest that *A. arktos* shows greatest affinity to habitat present at Best of All Lookout, specifically in areas of high leaf litter, bare soil, and rock ground cover and an intermediate abundance of shrubs and rainforest lomandra (*Lomandra spicata*). In contrast, Bilborough Court returned markedly fewer captures, with fewer shrubs, ferns, and no *L. spicata*, as well as less rock cover and low percentage grass cover. This implies that complex ground cover is preferred in meeting the foraging and refuge needs of *A. arktos*. Based on the diet of *A. arktos* as described by Gray et al. (2016), this complex, moist habitat type would support the marsupial's preferred arthropod prey.

Similar habitat requirements have been found in congeners such as yellow‐footed antechinus (*Antechinus flavipes*), which favors structurally complex habitat including areas of abundant fallen logs and rock crevices (Kelly & Bennett, 2008; Marchesan & Carthew, 2004; Stokes et al., 2004). Such microhabitat structure may provide small mammals, like *A. arktos*, reduced risked of predation, resources such as nesting sites, access to nearby foraging areas and/or shelter (Stokes et al., 2004). Other studies on smaller congeners such as agile antechinus (*A. agilis*) and the brown antechinus (*A. stuartii*) have found that dense and more complex understories account for greater capture rates. Fawn antechinus (*A. bellus*) in far north Northern Territory have been found to seek refuge in tree hollows and fallen logs, while also preferring a habitat with dense, shrubby understorey (Department of the Environment, 2017; Friend, 1985). Another northern species, the cinnamon antechinus (*A. leo*), is known to nest within tree hollows and occurs in greater abundance in conjunction with habitats exhibiting denser plant cover (Leung, 1999). Studies of silver‐headed antechinus (*A. argentus*) found that certain plant taxa such as Johnson's grass‐tree (*Xanthorrhoea johnsonii*) were strong predictors of *A. argentus* occurrence, as they are likely to be used for foraging and refugia (Mason et al., 2017). Southern congeners such as Tasmanian dusky antechinus (*A. swainsonii*) and mainland dusky antechinus (*A. mimetes*), the closest living relatives to *A. arktos*, have been known to show preference to habitats with a damp, low, and dense understorey of ferns and shrubs (Baker et al., 2015; Dickman, 1983; Poskitt et al., 1984). Interestingly, a parallel study focusing on best detection methods for rare mammal species made use of a conservation detection dog, which located *A. arktos* most frequently within rock fissures and crevices (Thomas et al.,^2020^). This provides further evidence for *A. arktos* having affinity with areas possessing higher percentages of rock cover.

Studies on various *Antechinus* and other small native Australian mammals have found that a dense understorey of shrubs, grasses, and the presence of rock crevices provides diurnal shelter for individuals from predation by invasive species (Bennett, 1993; Mason et al., 2017; Stokes et al., 2004). Disturbance by logging in areas such as Bilborough Court and Bar Mountain (see Section 4.2), not only modifies the structure of a species' habitat, altering vegetation and habitat characteristics, but can also facilitate invasions by exotic species (Holland & Bennett, 2007). Cats (*Felis catus*), dogs (*Canis familiaris*), and red foxes (*Vulpes vulpes*) were observed at three of four sites during the current study (CER, pers. obs.; Gray, Dennis, et al., 2017; McCormack, 2015). Notable differences in habitat structure between the studied sites are largely ground‐based, presumably providing better refugia for small, terrestrial mammals like *A. arktos* from these feral predators.

We also found significant differences in ground cover composition between Best of All Lookout and Bilborough Court in relation to *A. arktos* captures. Fine‐scale incidence of *A. arktos* was influenced greatest by percentage cover of leaf litter, then rock cover, and finally moss/lichen. According to Gray et al. (2016), like most antechinus and many dasyurids (e.g., see Dickman, 1983; Dickman, 2014; Dunlop, Rayner, & Doherty, 2017; Fisher & Dickman, 1993; Mason, Burwell, & Baker, 2015; Pearce, Burwell, & Baker, 2017; Scarff, Rhind, & Bradley, 1998), *A. arktos* is a generalist forager. However, Gray et al. (2016) also found *A. arktos* consumes considerably higher volumes of dipteran (fly) larvae and Diplopoda (millipedes) than its sympatric congener, *A. stuartii*. Dipteran larvae and Diplopoda are often uncovered in leaf litter and in wetter soil and provide ecosystem services in the form of detritus breakdown in rainforests (Leite‐Rossi, Saito, Cunha‐Santino, & Trivinho‐Strixino, 2016; Parker & Minor, 2015). Plausibly, *A. arktos* fossicks within detritus and wet, muddy slopes, foraging for these food sources. These areas best characterize the habitats where *A. arktos* were most often caught and would account for the observed strong relationship between capture location and leaf litter/rock cover.

The approach adopted in the present study of relating mammal captures to microhabitat spatial structure has permitted insights into the habitat requirements of a little‐known endangered mammal. This information will be useful in species habitat and distribution modeling, which will prioritize conservation planning and search efforts in the future.

## CONFLICT OF INTERESTS

None declared.

## AUTHOR CONTRIBUTION

Caitlin E. Riordan, hons student, completed the vegetation survey, analyzed the data, and wrote the manuscript. Coral Pearce assisted in experimental design, editing of manuscript, and data analysis. Andrew Baker supervised the project and assisted in experimental design, editing of manuscript, and data analysis. Bill McDonald contributed to fieldwork and vegetation survey. Ian Gynther was involved in expert consultation, data collection, and editing of manuscript.

## Figures and Tables

**Figure B1:**
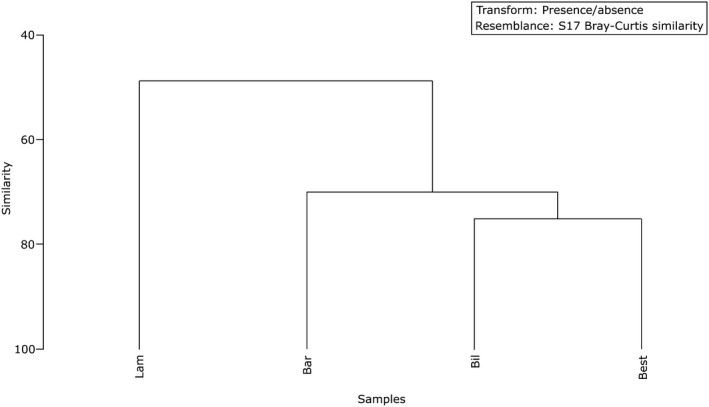
Dendrogram indicating hierarchical clustering of plant species assemblages from the four surveyed sites. The two clusters which are most similar are joined, forming a new cluster, represented here by Best of All Lookout (Best) and Bilborough Court (Bil). Deeper clustering groups incorporate Bar Mountain (Bar) and then Toolona Lookout, Lamington NP (Lam)

## Data Availability

Small mammal trapping data: Dryad, Vegetation survey data: Dryad, Both listed data sets are submitted to Dryad and are available under the https://doi.org/10.5061/dryad.qnk98sfbt.

## Appendix A Plant species assemblages

**Table A1:** Shared shrub species between known sites of *A. arktos* occurrence

Species	Bilborough Court	Bar Mountain	Best of All Lookout	Toolona Lookout
*Ardisia bakeri*	√		√	
***Cordyline rubra***	**√**	**√**	**√**	
*Cuttsia viburnea*				√
*Harpullia alata*			√	
*Hedycarya angustifolia*				√
*Helicia glabriflora*		√		
*Lenwebbia prominens*			√	
*Melicope hayesii*		√		
*Myrsine subsessilis* subsp*. subsessilis*	√	√	√	
*Pilidiostigma glabrum*			√	
***Pittosporum multiflorum***	√	√	√	
*Pittosporum oreillyanum*				√
***Psychotria simmondsiana***	√	√	√	√
*Rhodamnia maideniana*	√		√	
*Solanum inaequilaterum*		√		
*Tasmannia insipida*				√
***Triunia youngiana***	√	√	√	√
***Wilkiea huegeliana***	√	√	√	

Note

List is in alphabetical order, and most common (three or more shared) species among sites are highlighted in bold.

**Table A2:** Shared tree species between known sites of *A. arktos* occurrence

Species	Bilborough Court	Bar Mountain	Best of All Lookout	Toolona Lookout
*Acacia melanoxylon*	*√*	*√*		
***Ackama paniculosa***	*√*	*√*	*√*	*√*
*Acmena ingens*	*√*		*√*	
*Acmena smithii*		*√*	*√*	
*Acradenia euodiiformis*		*√*		
*Acronychia octandra*			*√*	
*Alectryon subcinereus*		*√*		
*Callicoma serratifolia*	*√*			*√*
***Cryptocarya foveolata***	*√*	*√*	*√*	*√*
*Cupaniopsis baileyana*		*√*		
*Cupaniopsis flagelliformis var. australis*		*√*		
*Daphnandra tenuipes*			*√*	*√*
*Diploglottis australis*	*√*			
***Doryphora sassafras***	*√*	*√*	*√*	*√*
***Duboisia myoporoides***	*√*	*√*	*√*	
***Dysoxylum fraserianum***	*√*	*√*	*√*	
*Dysoxylum rufum*			*√*	
*Emmenosperma alphitonioides*		*√*		
*Endiandra crassiflora*		*√*		
***Karrabina benthamiana***	*√*	*√*	*√*	
*Nothofagus moorei*		*√*		*√*
***Orites excelsus***	*√*	*√*	*√*	*√*
*Pennantia cunninghamii*		*√*	*√*	
***Polyosma cunninghamii***	*√*	*√*	*√*	*√*
*Polyscias murrayii*		*√*		
***Quintinia verdonii***	*√*	*√*	*√*	*√*
*Quintinia sieberi*			*√*	*√*
***Sarcopteryx stipata***	*√*	*√*	*√*	
*Sloanea australis* subsp. *australis*		*√*		
*Symplocos baeuerlenii*			*√*	
***Syzygium crebrinerve***	*√*	*√*	*√*	*√*
*Zanthoxylum brachyacanthum*			*√*	

Note

List is in alphabetical order, and most common (three or more shared) species among sites are highlighted in bold.

**Table A3:** Shared vines, liana, and epiphytic plant species between known sites of *A. arktos* occurrence

Species	Bilborough Court	Bar Mountain	Best of All Lookout	Toolona Lookout
***Asplenium australasicum***	*√*	*√*	*√*	*√*
***Austrosteenisia glabristyla***	*√*	*√*	*√*	
*Berberidopsis beckleri*		*√*		
*Carronia multisepalea*	*√*			
***Cephalaralia cephalobotrys***	*√*	*√*	*√*	*√*
*Dendrobium falcorostrum*				*√*
***Fieldia australis***		*√*	*√*	*√*
*Geitonoplesium cymosum*	*√*	*√*		
***Hibbertia scandens***	*√*	*√*	*√*	
*Marsdenia rostrata*		*√*		
***Melodinus australis***	*√*	*√*	*√*	
***Microsorum scandens***	*√*	*√*	*√*	*√*
***Palmeria reticulata***	*√*	*√*	*√*	
***Palmeria foremanii***	*√*	*√*	*√*	
*Pandorea floribunda*		*√*		
***Pararistolochia laheyana***	*√*	*√*	*√*	*√*
***Parsonsia fulva***	*√*	*√*	*√*	
*Parsonsia induplicata*		*√*		
*Parsonsia tenuis*				*√*
*Petermannia cirrhosa*			*√*	
***Pothos longipes***	*√*	*√*	*√*	*√*
***Ripogonum discolor***	*√*	*√*	*√*	
*Ripogonum fawcettianum*	*√*			*√*
*Rubus nebulosus*		*√*	*√*	
*Sarcopetalum harveyanum*			*√*	
***Smilax australis***	*√*	*√*	*√*	

Note

List is in alphabetical order, and most common (three or more shared) species among sites are highlighted in bold.

**Table A4:** Shared palm species between known sites of *A. arktos* occurrence

Species	Bilborough Court	Bar Mountain	Best of All Lookout	Toolona Lookout
***Archontophoenix cunninghamiana***	*√*	*√*	*√*	*√*
*Calamus muelleri*	*√*		*√*	
*Cordyline stricta* (grouped as palm in analysis)				*√*
***Linospadix monostachya***	*√*	*√*	*√*	*√*

Note

List is in alphabetical order, and most common (three or more shared) species among sites are highlighted in bold.

**Table A5:** Shared fern, tree fern, forb, and grass species between known sites of *A. arktos* occurrence

Species	Bilborough Court	Bar Mountain	Best of All Lookout	Toolona Lookout
***Arthropteris beckleri***	*√*	*√*	*√*	
*Arthopteris tenella*		*√*	*√*	
***Blechnum patersonii***		*√*	*√*	*√*
*Blechnum wattsii*				*√*
*Cyathea australis*				*√*
***Cyathea leichhardtiana***	*√*	*√*	*√*	*√*
*Dianella caerulea*				*√*
*Doodia* sp.			*√*	
*Drymophila moorei*				*√*
*Elatostema stipitatum*			*√*	*√*
***Helmholtzia glabberima***		*√*	*√*	*√*
***Lastreopsis* spp**.	*√*	*√*	*√*	*√*
***Lomandra spicata***	*√*	*√*	*√*	*√*
***Pellaea falcata***	*√*	*√*	*√*	*√*
*Vittaria* sp.				*√*

Note

List is in alphabetical order, and most common (three or more shared) species among sites are highlighted in bold.

## Appendix C Pairwise PERMANOVA results

**Table C1:** PERMANOVA comparison of vegetation and habitat structure between trap lines for, Best of All Lookout (a), Bilborough Court (b), Springbrook National Park, and Bar Mountain (c), Border Ranges National Park

Group	*t*‐value	*p* (perm) value	Group	*t*‐value	*p* (perm) value
1a, 1b	2.40	.0009	4a, 3c	2.85	.0003
1a, 2b	2.40	.0004	4a, 4c	2.97	.0001
1a, 3b	5.75	.0004	1b, 3b	4.01	.0004
1a, 4b	6.18	.0001	1b, 4b	4.27	.0001
1a, 1c	2.90	.0003	1b, 1c	1.66	.0164
1a, 2c	3.20	.0003	1b, 2c	2.38	.0041
1a, 3c	4.42	.0004	1b, 3c	1.76	.0125
1a, 4c	3.26	.0003	1b, 4c	2.53	.0014
2a, 1b	1.75	.0312	2b, 3b	5.15	.0003
2a, 2b	2.15	.0028	2b, 4b	5.35	.0003
2a, 3b	4.13	.0002	2b, 1c	1.99	.001
2a, 4b	4.41	.0002	2b, 2c	2.19	.0058
2a, 1c	2.44	.003	2b, 3c	2.75	.0004
2a, 2c	2.63	.0004	2b, 4c	2.26	.0011
2a, 3c	3.03	.0002	3b, 1c	4.33	.0005
2a, 4c	2.85	.0006	3b, 2c	4.69	.0003
3a, 3b	5.18	.0002	3b, 3c	4.71	.0002
3a, 4b	5.53	.0004	3b, 4c	5.35	.0005
3a, 1c	2.25	.001	4b, 1c	4.13	.0002
3a, 2c	3.02	.0005	4b, 2c	4.36	.0005
3a, 3c	2.75	.0008	4b, 3c	4.78	.0003
3a, 4c	3.12	.0003	4b, 4c	5.00	.0002
4a, 2b	1.64	.0356	1c, 2c	1.76	.0207
4a, 3b	4.80	.0001	1c, 4c	2.00	.0063
4a, 4b	5.15	.0002	2c, 3c	2.54	.0019
4a, 1c	2.24	.0004	3c, 4c	2.91	.0004
4a, 2c	2.92	.0001			
4a, 4b	5.15	.0002			

Note

Only significant *p*‐values (<.05) are displayed to represent structural habitat differences between lines. Line 1 (traps 1–25), line 2 (traps 26–50), line 3 (traps 51–75), and line 4 (traps 76–100).

**Table C2:** PERMANOVA comparison of percentage ground cover between trap lines for Best of All Lookout (a), Bilborough Court (b), Springbrook National Park, and Bar Mountain (c), Border Ranges National Park

Group	*t*‐value	*p* (perm) value	Group	*t*‐value	*p* (perm) value
1a, 1b	1.56	.0324	2a, 4c	2.03	.004
1a, 4b	1.73	.0341	3a, 1b	1.60	.0172
1a, 1c	2.13	.0039	3a, 3b	1.77	.0161
1a, 2c	2.08	.0019	3a, 4b	1.88	.0167
1a, 4c	2.05	.0025	3a, 1c	2.05	.0083
2a, 1b	1.63	.0196	3a, 2c	1.84	.0069
2a, 3b	1.88	.0074	3a, 4c	1.59	.0452
2a, 4b	1.98	.0031	4a, 4b	1.68	.0258
2a, 1c	2.10	.0036	1b, 4b	1.97	.0079
2a, 2c	1.95	.0028	1b, 1c	1.91	.0137
2a, 3c	1.64	.0281	1b, 2c	1.65	.0286

Note

Only significant *p*‐values (<.05) are displayed to represent structural habitat differences between lines. Line 1 (traps 1–25), line 2 (traps 26–50), line 3 (traps 51–75), and line 4 (traps 76–100).

**Table C3:** PERMANOVA comparison of habitat structure between trap lines for both Best of All Lookout (a) and Bilborough Court (b), Springbrook National Park

Group	*t*‐value	*p* (perm) value
1a, 1b	2.40	.0011
1a, 2b	2.40	.0001
1a, 3b	5.75	.0003
1a, 4b	6.18	.0003
2a, 1b	1.75	.0325
2a, 2b	2.14	.0037
2a, 3b	4.13	.0002
2a, 4b	4.41	.0003
3a, 3b	5.18	.0002
3a, 4b	5.53	.0003
4a, 2b	1.63	.0331
4a, 3b	4.08	.0002
4a, 4b	5.15	.0002
1b, 3b	4.01	.0003
1b, 4b	4.27	.0001
2b, 3b	5.14	.0003
2b, 4b	5.34	.0002

Note

Only significant *p*‐values (<.05) are displayed to represent structural habitat differences between lines. Line 1 (traps 1–25), line 2 (traps 26–50), line 3 (traps 51–75), and line 4 (traps 76–100).

**Table C4:** Pairwise PERMANOVA comparison of ground cover percentages between trap lines for both Best of All Lookout (a) and Bilborough Court (b), Springbrook National Park

Group	*t*‐value	*p* (perm) value
1a, 1b	1.56	.0304
1a, 4b	1.73	.0343
2a, 1b	1.63	.0215
2a, 3b	1.88	.0088
2a, 4b	1.98	.0029
3a, 1b	1.59	.0167
3a, 3b	1.77	.0165
3a, 4b	1.88	.0187
4a, 4b	1.68	.0267
1b, 4b	1.97	.0086

Note

Only significant *p*‐values (<.05) are displayed to represent differences in ground cover attributes between lines. Line 1 (traps 1–25), line 2 (traps 26–50), line 3 (traps 51–75), and line 4 (traps 76–100).
